# Curcumin: a potential anti-photoaging agent

**DOI:** 10.3389/fphar.2025.1559032

**Published:** 2025-05-06

**Authors:** Yuhong Nie, Yiming Li

**Affiliations:** Department of Dermatology, Institute of Traditional Chinese Medicine of Sichuan Academy of Chinese Medicine Sciences, Chengdu, China

**Keywords:** curcumin, photoaging, signaling pathways, nanoparticle formulations, delivery systems

## Abstract

Turmeric, also referred to as *Curcuma longa*, is a commonly used spice, recognized for its demonstrated effects in reducing inflammation, combating microbes, providing antioxidant benefits, slowing the aging process, and exhibiting anticancer potential. The process of skin aging is intricate, with ultraviolet radiation being a significant extrinsic factor. Increasing evidence suggests that curcumin, the active component of turmeric, can prevent ultraviolet radiation-induced skin photoaging and related inflammation. Its effects include inhibition of melanin production, wrinkle reduction, antioxidant and anti-inflammatory actions. This review primarily focuses on the specific signaling pathways involved in skin photoaging and the mechanisms by which curcumin mitigates photoaging. Key topics include the antioxidant and anti-inflammatory properties of curcumin, regulation of matrix metalloproteinase, regulation of autophagy and apoptosis, improvement of pigmentation, and regulation of microbial balance. Additionally, addressing the critical issue of curcumin’s low bioavailability, the review summarizes the latest advancements in curcumin formulation improvements. This article aims to provide a comprehensive overview of curcumin’s progress of skin photoaging research and offer evidence for its further clinical application in dermatological treatments. The review contributes to a deeper understanding of the potential molecular mechanisms of curcumin in combating photoaging and presents new insights for the development of curcumin-based anti-photoaging products.

## 1 Introduction

### 1.1 Skin photoaging

**FIGURE 1:**
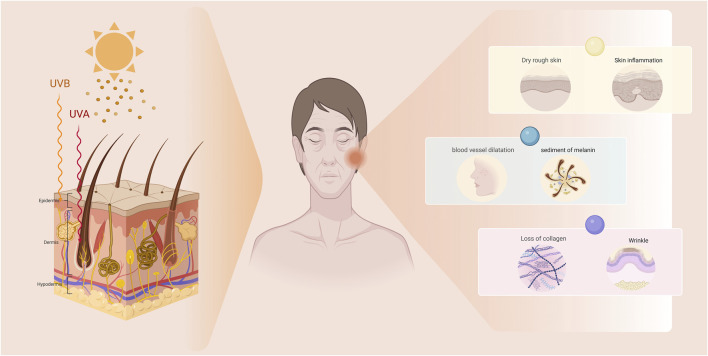
The manifestations of photoaging in the skin. Photoaged skin primarily manifests as dryness, roughness, deepening wrinkles, skin laxity, telangiectasia, and hyperpigmentation.

Human skin is a complex and dynamic organ with a highly specialized structure as shown in Figure 1. It consists of various cell types and different functional areas. Among them, the dermis, which provides structural support, nutrient supply and circulatory function to the skin, changes in all layers of the skin with age, which is reflected in the alteration of skin structure and function (McLafferty et al., 2012; Tobin, 2017). Skin aging can be categorized into two types: intrinsic aging and extrinsic aging (Poljšak et al., 2012). Intrinsic aging is driven by inevitable physiological processes within the body, progressing slowly and being difficult to modulate. In contrast, extrinsic aging is significantly influenced by external factors and lifestyle habits, with key accelerators including ultraviolet radiation (UVR), air pollution, psychological stress, and smoking (Koohgoli et al., 2017). Among these external factors, ultraviolet radiation is the primary contributor to skin aging, leading to what is known as photoaging (Bosch et al., 2015). Ultraviolet radiation consists of three wavelengths: Ultraviolet A (UVA) (320–400 nm), Ultraviolet B (UVB) (280–320 nm), and Ultraviolet C (UVC) (100–280 nm) (Kim B-H. et al., 2015) is entirely absorbed by the Earth’s atmosphere, leaving only UVA and UVB to reach the surface (with UVA accounting for approximately 95% and UVB about 5%). Nearly all UVB photons are captured by large molecules within the epidermis, while UVA rays can permeate the epidermis, reaching into the dermal layer (Holick, 2016). These two types of UV radiation are the major causes of skin damage and accelerated aging. Although UVB has higher energy and is the main cause of sunburn, its shorter wavelength means it is mostly absorbed by the epidermis, with only a small fraction penetrating the dermis. On the other hand, UVA can penetrate deeper into the skin layers and is therefore widely recognized as the key factor in photoaging (Kammeyer and Luiten, 2015). Photoaging typically manifests as dryness, roughness, deepening wrinkles, skin laxity, vascular changes, and hyperpigmentation in sun-exposed areas (Zouboulis and Hoenig, 2019).

### 1.2 Curcumin and biological effects

Diet plays a crucial role in the aging process. Recent studies have demonstrated that dietary polyphenols, such as phenolic acids and flavonoids, can delay aging by mitigating oxidative stress, modulating signaling pathways, and influencing gene expression (Samarghandian et al., 2017). Curcumin (CUR) is an active polyphenol extracted from the rhizomes of turmeric, a member of the zingiberaceae family. Historically, curcumin has been highly regarded for its potent anti-inflammatory properties. Modern medicine has extensively validated its therapeutic potential, leading to its widespread use in the prevention and treatment of various conditions, including inflammatory diseases, infectious diseases, respiratory disorders, cardiovascular diseases, neurological disorders, cancers, mental health issues, and metabolic disorders (Hatcher et al., 2008).

Curcumin, or (1E,6E)-1,7-bis (4-hydroxy-3-methoxyphenyl)-1,6-heptadiene-3,5-dione, is a symmetric molecule known as diferuloyl methane (MW368.38 g mol^−1^) (Priyadarsini, 2014). Its structure includes two omethoxyphenol groups linked by a seven-carbon chain with an α,β-unsaturated diketone, facilitating electron transfer reactions (Moustapha et al., 2015; Sala de Oyanguren et al., 2020). Curcumin exhibits high lipophilicity, and according to the study by Moustapha team (Moustapha et al., 2015), it is rapidly internalized by cells at a ratio of 1/20 relative to its external concentration. Additionally, curcumin has been detected within the endoplasmic reticulum and lysosomes.

Due to its biphasic dose-response profile in inducing stress response pathways, curcumin is categorized as a hormetin (Demirovic and Rattan, 2011). According to Rainey (Rainey et al., 2015), extremely low doses (≤1 μM) of curcumin act as potent antioxidants. However, at moderate doses (5–10 μM), curcumin primarily functions as an autophagy inducer, which is associated with its ability to reduce cytoplasmic protein acetylation and induce cell cycle arrest (Pietrocola et al., 2012). Finally, at higher doses (exceeding 25 μM), curcumin induces cell death, with all experiments conducted over a 48-h period. This paper further summarizes the mechanisms of curcumin in combating photoaging and the advancements in research on this topic.

## 2 Materials and methods

### 2.1 Search strategy

An online literature search was carried out at PubMed, Web of Science, Embase, Wanfang Data and CNKI, covering 2014 until April 2025. The following keywords were used: “curcumin” and “skin photoaging”, or “anti-oxidation”, or “anti-inflammation”, or “matrix metalloproteinases”, and “apoptosis”, or “autophagy”, or “melanogenesis”, or “microorganism”, or “formulation”, or “bioavailability”, or “drug delivery systems, or “nanoparticles”. The references of all retrieved articles were also reviewed to include relevant literature. The same selection criteria were applied to all databases, and duplicate studies across databases were identified and removed using Zotero and manual cross-checking.

### 2.2 Selection criteria

The inclusion criteria are as follows: curcumin and its compounds are the subjects of the study; the diseases and physiological processes targeted by curcumin interventions are related to aging and photoaging; the study design is clear, and the study results involve the exploration of relevant mechanisms; the latest research on curcumin’s clinical applications and formulations is included; the literature was published within the past 10 years, unless it holds significant historical value; the research must explicitly describe molecular mechanisms or signaling pathways and provide detailed information on curcumin formulations or delivery systems (e.g., nanoparticles, liposomes) and their effects on bioavailability or efficacy.

The exclusion criteria are as follows: studies in literature reviews with unclear subjects, methods, or mechanisms; studies with a very small sample size; studies with poor methodology, unreliable results, or low quality; duplicate publications of the same research content; articles such as conference abstracts, editorials, or opinion pieces that do not provide significant original data; unpublished or non-peer-reviewed studies.

During the collation process, standardized data extraction forms were used to ensure consistency in research data. Additionally, two researchers independently screened and analyzed all the literature to ensure the reliability of the study.

## 3 Mechanism of anti-photoaging effect of curcumin

### 3.1 Anti-oxidation

Reactive oxygen species (ROS) drive skin photoaging by oxidizing collagen, elastin, and DNA, leading to impaired barrier function and wrinkle formation. UV radiation activates three key oxidative stress pathways: the epidermal growth factor receptor (EGFR)-mitogen-activated protein kinase (MAPK) cascade, nuclear factor kappa B (NF-κB)-mediated inflammation, and the nuclear factor erythroid 2-related factor 2(Nrf2)/antioxidant response element (ARE) axis. Under physiological conditions, Nrf2 binds to Kelch-like ECH-associated protein 1(Keap1) in the cytoplasm, undergoing ubiquitination and degradation. ROS or UV exposure disrupts Keap1’s conformation, releasing Nrf2 to translocate into the nucleus, where it activates antioxidant enzyme genes to establish cellular defense systems (Kovac et al., 2015; Krajka-Kuźniak et al., 2017).

Curcumin targets the Keap1-Nrf2 pathway by modifying Keap1’s thiol groups, destabilizing the Keap1-Nrf2 complex, and blocking Nrf2 ubiquitination. This promotes Nrf2 nuclear translocation and ARE binding, upregulating antioxidant enzymes such as Superoxide Dismutase 1(SOD1), Heme Oxygenase-1(HO-1), and Glutathione Peroxidase (GPx) (Wang et al., 2020; Lin et al., 2019). Its effects are dose-dependent: low concentrations (5–20 μM) enhance antioxidant capacity (e.g., 20 μM curcumin increased SOD activity by 47% in RAW264.7 cells) (Lin et al., 2019); moderate concentrations (20–60 μM) induce cell cycle arrest and suppress telomerase activity; high concentrations (≥80 μM) disrupt cellular structures and promote apoptosis (Mollazade et al., 2013). Topically applied curcumin (5 mg in Vaseline cream) significantly elevates Nrf2, HO-1, and SOD levels in mouse skin (WU et al., 2023), while nanocarrier-loaded curcumin (10 μg/mL) synergistically upregulates Glutathione Peroxidase one and Nrf2 expression (Liakopoulou et al., 2025). Additionally, curcumin restores UV-induced mutant p53 function by modulating B-cell lymphoma 2 Associated X Protein/B-cell lymphoma 2(Bax/Bcl-2)balance, reversing apoptosis resistance and improving mitochondrial homeostasis (Rebel et al., 2012).

In summary, curcumin orchestrates a multi-dimensional anti-photoaging network through Keap1-Nrf2 pathway regulation, integrating antioxidant defense (low doses), cell cycle intervention (moderate doses), and pro-apoptotic effects (high doses). It concurrently repairs DNA damage, inhibits telomerase activity, and enhances mitochondrial stability. Future research should focus on elucidating its cross-scale mechanisms (molecular-cellular-tissue) and synergistic interactions with other antioxidants to optimize its clinical application in photodamage prevention and treatment.

### 3.2 Anti-inflammation

**FIGURE 2:**
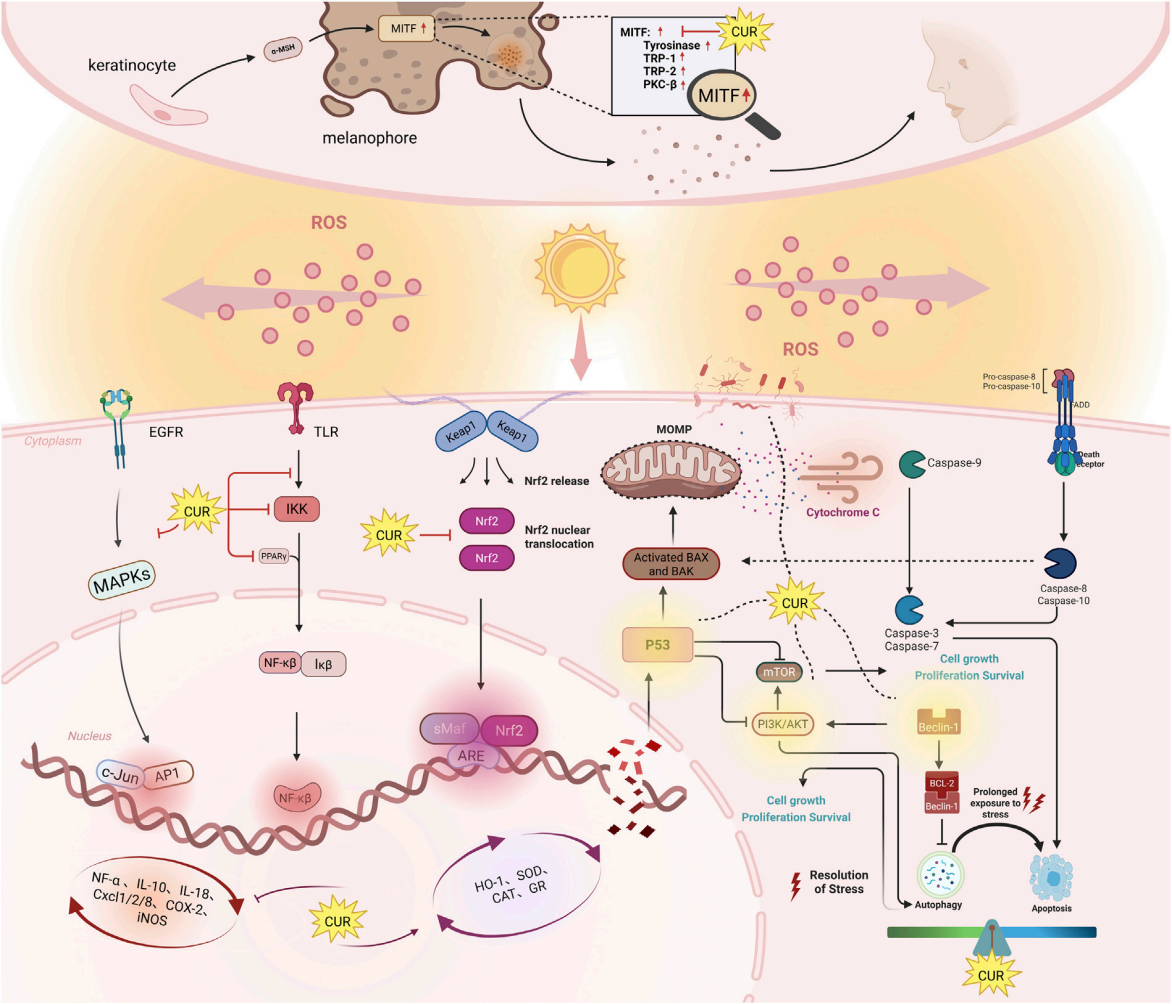
The mechanisms of anti-photoaging effects of curcumin. This diagram shows six key signaling pathways. Nrf2: When ROS levels rise, Nrf2 detaches from the Keap1 complex and enters the nucleus. There, it activates antioxidant enzymes like heme oxygenase-1, SOD, CAT, and GR, which protect against oxidative stress. NF-κB: Curcumin reduces inflammation by activating PPARγ, blocking TLR activation, and inhibiting IKK and IκB phosphorylation. This prevents NF-κB from moving to the nucleus, reducing inflammation. MAPK: Curcumin blocks the UVB-induced phosphorylation of p38 and JNK, stopping the activation of downstream molecules. This suppresses inflammation and apoptosis, preventing skin aging by inhibiting MMPs like MMP-1 and MMP-3. Autophagy: Curcumin regulates autophagy via Beclin-1, p53, and PI3K/AKT/mTOR. At low doses, it helps cell survival; at higher doses, it triggers autophagy-related cell death and cycle arrest. Apoptosis: Curcumin promotes apoptosis in damaged or cancerous cells by increasing caspase-3 and Bax expression, and inhibiting anti-apoptotic proteins like Bcl-2 and PI3K/AKT. In healthy cells, it prevents excessive apoptosis, protecting against UV-induced skin damage. Symbols: Pointed arrow (↓): indicates promotion. The bold arrow (⊥): indicates inhibition.

Chronic inflammation is closely linked to the progression of skin photoaging. UVR-induced DNA damage and alterations in the extracellular matrix (ECM) disrupt homeostasis and trigger cellular stress, leading to the activation of inflammatory responses in the skin as shown in Figure 2. As previously mentioned, the NF-κB and p38 MAPK pathways promote the expression of cytokines and chemokines, which are crucial for the recruitment of cells during the progression of inflammation (Cavinato and Jansen-Dürr, 2017; Hasegawa et al., 2016; Fitsiou et al., 2021). NF-κB is identified as a transcription factor with a pivotal role in managing inflammation and immune responses, as well as in the transcriptional control of various chemokines and cytokines. It is instrumental in the regulation of immune reactions, inflammation, cell proliferation, apoptosis, and cellular responses (Zinatizadeh et al., 2021).

Curcumin exerts anti-inflammatory effects by targeting key nodes of the NF-κB signaling pathway. In the initial phase (1.5–5 μM), it activates peroxisome proliferator-activated receptor γ(PPARγ) receptors and inhibits toll-like receptor 4/myeloid differentiation primary response 88 (TLR4/MyD88) complex formation, blocking inhibitor of nuclear factor kappa-B kinase subunit β(IKKβ) phosphorylation (IC50 = 5.2 μM). This stabilizes Inhibitor of NF-κB (IκBα) and reduces NF-κB nuclear translocation by 76% (Wang et al., 2018; Nguyen et al., 2022). At elevated concentrations (5–10 μM), curcumin directly binds to the DNA-binding domain of the NF-κB p65 subunit (Kd = 2.3 nM) and the adenosine triphosphate (ATP)pocket of IKKβ (ΔG = −7.6 kcal/mol), exerting steric hindrance to impair their functions (Wang et al., 2018; Martín-Vázquez et al., 2023).

When concentrations reach 10–20 μM, its effects extend to downstream inflammatory mediators, reducing cyclooxygenase-2(COX-2), interleukin-6(IL-6), and tumor necrosis factor-α(TNF-α) expression by over 60% while upregulating the anti-apoptotic protein Bcl-2 (Lee et al., 2020). Notably, at 6 μM, curcumin synergistically reduces interleukin-1β(IL-1β)/IL-6 secretion by 50% through inhibition of extracellular signal-regulated kinases/c-Jun N-terminal kinases (ERK/JNK) phosphorylation (EC50 = 8.4 μM) and targeting of the signal transducer and activator of transcription 3 src homology 2 (STAT3 SH2) domain (ΔG = −7.1 kcal/mol) (Peng et al., 2021; Martín-Vázquez et al., 2023).

This gradient mechanism—ranging from low-concentration signaling blockade to high-concentration multi-pathway synergy—exhibits concentration-dependent anti-inflammatory activity in LPS-stimulated HaCaT keratinocytes (1.5–20 μM), with no cytotoxicity observed below 6 μM (Nguyen et al., 2022). Experimental data reveal that curcumin achieves comprehensive pathway intervention through precise concentration windows, spanning upstream activation suppression, midstream nuclear translocation blockade, and downstream gene expression regulation.

Curcumin has garnered attention for its remarkable anti-inflammatory effects, primarily achieved by modulating key signaling pathways such as NF-κB, MAPK, and activator protein 1 (AP-1). Although existing research has extensively described curcumin’s effects on these pathways, its mechanisms of action in the context of chronic UV-induced damage remain inadequately understood. To assess the safety, efficacy, and optimal dosage of curcumin, particularly for skin protection, more long-term *in vivo* studies and clinical trials are necessary. Moreover, exploring the synergistic effects of curcumin with other therapeutic approaches is crucial. Future research should also investigate emerging areas of application, such as epigenetic regulation and the impact on the skin microbiome, to uncover its broader therapeutic potential.

### 3.3 MMPs regulation

Collagen is the predominant insoluble fibrous protein found in the ECM and connective tissues, primarily synthesized by fibroblasts within the dermis (Wen et al., 2012). Matrix metalloproteinases (MMPs) are zinc-dependent endopeptidases with broad substrate specificity, responsible for the degradation of various ECM components as shown in Figure 2 (Pittayapruek et al., 2016; Kim et al., 2011). Exposure to UVA and UVB radiation induces oxidative stress in human skin, leading to chronic genetic damage, upregulation of AP-1 activity, and increased Matrix metalloproteinases (MMP) expression. In addition to AP-1, ROS generation also induces NF-κB-mediated transcriptional activation. It has been reported that the activation of NF-κB is responsible for the upregulation of MMPs, such as MMP-1 and MMP-3, in dermal fibroblasts (Vicentini et al., 2011; Lee et al., 2012). Consequently, both AP-1 and NF-κB are involved in the process of photoaging.

Curcumin effectively counteracts MMP-mediated skin photoaging through multi-target mechanisms. Its core actions involve: (1) Direct binding to the catalytic domain of matrix MMP-9(Kd = 8.4 nM) and active sites of MMP-1/3, with Ce6-diPEG-curcumin conjugates (200 nM) combined with photodynamic therapy reducing UVB-induced MMP-2 expression by 67% (Thapa Magar et al., 2023; Liu et al., 2024); (2) Dual blockade of the NF-κB/AP-1 signaling axis via inhibition of IKKβ phosphorylation (IC50 = 5.8 μM) to reduce NF-κB nuclear translocation, coupled with suppression of JNK/ERK phosphorylation (EC50 = 7.2 μM) to impede c-Jun/Fos complex formation, thereby synergistically downregulating MMP-1/3/9 transcription (Hwang et al., 2013; Liu et al., 2024); (3) Activation of the transforming growth factor-β/small mothers against decapentaplegic 2/3 (TGF-β/Smad2/3) pathway (5 μM) to promote collagen synthesis, while scavenging ROS through the Nrf2/HO-1 axis, reducing UVB-induced oxidative damage by 52% (Liu et al., 2018; Yuan et al., 2017).

Dose-response analyses reveal gradient regulatory characteristics of curcumin within the 5–30 μM range: lower concentrations (5–10 μM) preferentially repair the extracellular matrix, whereas therapeutic concentrations (20–30 μM) achieve potent anti-photoaging effects through multi-pathway synergy. Recent photodynamic strategies further enhance efficacy via tissue-targeted delivery of Ce6 conjugates, evidenced by a 3-fold increase in hepatic/pulmonary fluorescence intensity (Thapa Magar et al., 2023).

Future research should focus on deciphering curcumin’s regulatory network on MMP/tissue inhibitors of metalloproteinases (TIMP) dynamic balance and developing nano-delivery systems to overcome its bioavailability limitations (current oral bioavailability <1%), thereby accelerating clinical translation. This multimodal mechanism—encompassing anti-inflammatory, antioxidant, and matrix remodeling properties—establishes curcumin’s core advantages as a natural photoprotective agent.

### 3.4 Autophagy interference

Emerging evidence highlights the critical role of autophagy modulation in counteracting UV-induced photoaging, with curcumin demonstrating dose-dependent bidirectional regulatory effects through targeted molecular pathways. Recent studies (Tsang et al., 2022; Zuo et al., 2021) reveal that autophagy mitigates UV-induced oxidative stress, DNA damage, and aberrant cell proliferation by orchestrating cellular repair and survival-death balance. Curcumin exerts context-specific autophagy regulation through dual mechanisms: At moderate concentrations (20–25 μM), it promotes cytoprotective autophagy via inhibition of the mechanistic target of rapamycin (mTOR)-AKT axis (Zhao et al., 2016), evidenced by decreased p-AKT, p-mTOR, and p-P70S6K expression, coupled with increased microtubule-associated protein 1A/1B-light chain 3- II/microtubule-associated protein 1A/1B-light chain 3-I (LC3-II/LC3-I) conversion. This facilitates cell cycle arrest at G_2_/M phase and enables cellular recovery, as observed in Huh-7 cells treated with 20 μM curcumin (Sala de Oyanguren et al., 2020). Conversely, higher concentrations (50–75 μM) trigger autophagic cell death through Beclin-1 activation and p53 modulation, independent of caspase pathways (Hasima and Ozpolat, 2014; Zhao et al., 2016). The compound’s therapeutic synergy is amplified when combined with photodynamic therapy-3.5 μM curcumin with light irradiation enhances autophagic flux while suppressing ERK/AKT-mediated oxidative stress, converting reversible cell cycle arrest to irreversible senescence (Niu et al., 2016). Notably, *in vivo* efficacy is maintained at 25 mg/kg dosage, significantly reducing melanoma growth through coordinated autophagy-proliferation regulation (Zhao et al., 2016).

Curcumin modulates photoaging through concentration-dependent crosstalk between autophagy regulators and stress-response pathways. It primarily inhibits the mTOR-AKT signaling axis to activate pro-survival autophagy at lower doses, while engaging Beclin-1 and p53 to drive autophagic cell death at higher concentrations. Downstream effects involve LC3-mediated autophagosome formation, G_2_/M phase arrest through cyclin regulation, and suppression of oxidative stress via ERK/AKT dephosphorylation. These multimodal actions position curcumin as a promising candidate for precision interventions in UV-induced skin damage and melanoma progression.

### 3.5 Apoptosis regulation

#### 3.5.1 Apoptosis promotion

Ultraviolet radiation disrupts epidermal homeostasis by concurrently activating pro-apoptotic pathways (e.g., p53/Bax upregulation, p38 MAPK activation) and pro-survival signals (e.g., COX-2-mediated proliferation) (Mlitz et al., 2016; Lee, 2019). Curcumin counteracts UV-induced pathological proliferation by selectively inducing apoptosis in photodamaged or malignant cells through dose-dependent mechanisms. At higher concentrations (10–100 μM), curcumin suppresses EGFR tyrosine kinase activity, blocking downstream phosphoinositide 3-Kinase (PI3K)/AKT survival signaling in melanoma cells (Chiu et al., 2022). Concurrently, it activates caspase-3 expression and enzymatic activity, amplifying apoptotic execution in SK-MEL-28 and A375 cells (Manica et al., 2023). These actions collectively reduce cell viability, inhibit migration, and eliminate premalignant clones, thereby mitigating UV-driven carcinogenesis.

#### 3.5.2 Apoptosis inhibition

In contrast, curcumin exhibits cytoprotective effects in non-cancerous contexts by suppressing UV-induced apoptosis at lower concentrations (0.1–10 μM). Pretreatment with 5 μM curcumin in human dermal fibroblasts (HDFs) significantly downregulates caspase-3 and upregulates anti-apoptotic Bcl-2, preserving cellular integrity under UVA stress (Liu et al., 2018). Furthermore, curcumin modulates adenosine metabolism by inhibiting CD39/CD73/adenosine deaminase (ADA) expression (0.1–10 μM), thereby attenuating immunosuppressive microenvironments in SK-Mel-28 cells (Manica et al., 2024). This dual regulation—balancing pro-survival signals and anti-inflammatory pathways (adenosine metabolism)—highlights its capacity to protect normal skin cells from UV-induced damage while maintaining tissue homeostasis.

This context-specific modulation enables curcumin to selectively eliminate damaged or cancerous cells while shielding healthy tissue, underscoring its potential as a dual-function agent against photoaging. Clinical translation, however, requires advanced delivery systems to enhance bioavailability and precision in targeting skin-specific pathways.

### 3.6 Melanogenesis reduction

Ultraviolet radiation stimulates melanogenesis through activation of the microphthalmia-associated transcription factor (MITF)-regulated pathway, promoting tyrosinase (TYR)-dependent melanin synthesis in melanosomes to protect against DNA damage, while excessive production leads to hyperpigmentation disorders (Kim et al., 2021). Curcumin and its derivatives suppress melanogenesis through dual strategies: direct tyrosinase inhibition and MITF-mediated transcriptional regulation. In α-melanocyte stimulating hormon (α-MSH)-stimulated B16F10 cells, curcumin (5–10 μM) and bisdemethoxycurcumin (5–10 μM) downregulate melanogenic genes, reducing melanin synthesis by 40%–60% (Jeon et al., 2023). Structurally optimized derivatives like CMC2.24 demonstrate enhanced efficacy, inhibiting tyrosinase activity in MNT-1 human melanoma cells at 5–25 μM without cytotoxicity (Goenka et al., 2021). Notably, curcumin’s effects are redox-dependent: at 10 μM, it suppresses baseline melanogenesis in B16F10 cells but exhibits biphasic behavior under oxidative stress—promoting melanin at low H_2_O_2_ (<0.3 mM) while inhibiting it at higher H_2_O_2_ levels (Wolnicka-Glubisz et al., 2015).

Curcumin targets melanogenesis by competitively binding tyrosinase to block catalytic activity and disrupting the MITF-TYR/Transient Receptor Potential (TRP) transcriptional axis. Future research must optimize curcumin’s bioavailability via nanoformulations and validate its dose-response relationships *in vivo*. Addressing these challenges will unlock its potential as a dual-function agent against photoaging and hyperpigmentation.

### 3.7 Restoring microbial homeostasis

The skin and gut microbiota collaboratively maintain cutaneous homeostasis through metabolic and immune interactions. Resident skin microbes secrete enzymes critical for barrier function: proteases facilitate stratum corneum renewal, lipases degrade surface lipids, and ureases regulate urea metabolism. Concurrently, bacteriocin production, quorum sensing, and pH modulation establish a microbial defense network n (Boxberger et al., 2021). The gut-skin axis enables bidirectional communication, where dysbiosis increases intestinal permeability, allowing bacterial metabolites (e.g., lipopolysaccharides) to accumulate in the skin via systemic circulation, disrupting epidermal differentiation and barrier integrity (Dinan and Cryan, 2017; Szántó et al., 2019). UVR disrupts microbial equilibrium through direct DNA damage and indirect release of pathogen-associated molecular patterns (PAMPs) and damage-associated molecular patterns (DAMPs), such as oxidized lipids and porphyrins, promoting pathogenic overgrowth (e.g., *Staphylococcus aureus*). These dysbiotic communities activate IL-17/TNF-α signaling, upregulate MMP-1/MMP-3 to degrade collagen, and suppress filaggrin and tight junction proteins, driving a pathological cascade of oxidative stress, chronic inflammation, and barrier dysfunction (Kim et al., 2021; Seo et al., 2023).

Curcumin addresses microbial dysregulation through localized and systemic mechanisms. Topically applied at concentrations ≥50 μM, its hydrophobic structure disrupts bacterial membrane integrity, inhibits virulence factor expression in pathogens like *S. aureus*, and enhances photodynamic antibacterial activity under blue light irradiation (Zheng et al., 2020). Systemically, curcumin modulates the NF-κB/AP-1 pathway to reshape gut microbiota composition, promoting the proliferation of short-chain fatty acid (SCFA)-producing bacteria while suppressing pathogenic colonization. This restoration of Th17/Treg immune balance is critical for mitigating systemic inflammation (Kasprzak-Drozd et al., 2024). SCFAs further alleviate cutaneous photoaging by activating G protein-coupled receptors (GPCRs), downregulating pro-inflammatory cytokines (e.g., IL-1β, IL-6), and inhibiting MMP-1/MMP-3 expression, thereby reducing collagen degradation (Peterson et al., 2019).

Emerging evidence highlights UV-induced microbial dysbiosis as a key driver of photoaging through oxidative stress, inflammation, and barrier impairment. Curcumin demonstrates therapeutic potential via its dual antimicrobial and immunomodulatory properties. Future research should prioritize identifying photoaging-specific microbial biomarkers, optimizing curcumin’s topical delivery systems to enhance bioavailability, and elucidating the transport mechanisms of microbial metabolites across the gut-skin axis.

## 4 Limitations and countermeasures of curcumin in clinical application

### 4.1 Limitations of curcumin application

However, the application of curcumin is limited by its physicochemical properties, particularly its low water solubility and poor intestinal permeability. Addressing these challenges is essential for improving its efficacy. Extensive research has focused on this issue, with nanotechnology emerging as a promising solution to enhance curcumin’s bioavailability. Encapsulation of curcumin in nanoparticles can help overcome delivery obstacles, thereby improving its bioavailability and stability. This review explores the mechanisms through which curcumin combats photoaging, recent advancements in understanding the associated signaling pathways, and summarizes the latest research aimed at enhancing curcumin’s bioavailability. These insights provide new perspectives on the application of curcumin in skin-related diseases.

Curcumin has not yet been approved as a therapeutic drug, mainly due to its poor water solubility, inadequate intestinal absorption, and rapid metabolism, which significantly limit its effectiveness in the human body (Wen et al., 2023). After oral administration, the majority of curcumin is excreted as metabolites, with only a small fraction entering the bloodstream, at concentrations well below those required to inhibit most of its anti-inflammatory targets. Curcumin is a diketone pigment composed of two ortho-methylated phenols and a β-diketone functional group, with the β-diketone portion believed to be responsible for its instability, rapid degradation, and low bioavailability (Vargas-Mendoza et al., 2022).

### 4.2 Research progress on improvement of curcumin clinical applications

In recent years, the preparation of curcumin has been continuously explored. Recent clinical studies demonstrate curcumin’s dual role in skin repair. A 2022 trial (N = 60) on photoaging management showed that a 4-week regimen combining oral 70 mg curcumin with topical 0.02% curcumin cream significantly improved skin firmness (+11.2% vs. 5.5%, p < 0.01), elasticity (+12.7%, assessed by Cutometer^®^), and reduced forehead wrinkle volume (−16.5%, measured via Visioface^®^) compared to topical-only treatment. It also enhanced skin barrier function (10.8% reduction in transepidermal water loss [TEWL], 3.5% increase in hydration) and increased collagen density (validated by Dermascan^®^ ultrasound imaging) (Di Lorenzo et al., 2023). In a 2025 double-blind trial (N = 52) on breast cancer radiotherapy patients, topical 2% curcumin gel applied for 4 weeks significantly outperformed placebo in reducing erythema incidence (3.7% vs. 96%, p < 0.01), achieving pain-free rates (70.4% vs. 28%), and lowering irritant reactions (37% vs. 84%, p < 0.01), with no additional risk of itching or dryness. These studies validate the clinical potential of curcumin’s “oral-topical” strategy (Heydari et al., 2025).

Carrier design critically impacts drug penetration and stability. Poly (lactic-co-glycolic acid)/hyaluronic acid (PLGA/HA) microneedles using chitosan-PLGA composite nanocarriers increased curcumin transdermal rate by 4.2-fold and extended duration to 2 months (Chen et al., 2023). Cur-Res SLNs, formulated with Compritol 888 ATO lipid cores (average particle size 180.2 nm), achieved encapsulation efficiencies of 92% (CUR) and 62.8% (Res), with over 70% of the drug bound to the skin in transdermal experiments. Nanoencapsulation accelerated Res release by 5-fold compared to free forms. Comparative studies demonstrated that aqueous-based formulations underperformed lipid carriers (ΔVISIA scores: −12.4vs. −19.7), highlighting carrier selection as pivotal for efficacy (Palliyage et al., 2021). It should be noted that some trials failed to detect any beneficial effects of curcumin, and some trials also failed in elderly subjects and patients with atopic dermatitis, despite the use of significant doses for weeks or months, presumed to be related to bioavailability (Lagoa et al., 2025). In terms of safety, the optimization of the delivery system resulted in a significant reduction of irritation in the compounded formulation, with the main adverse effects identified in the trials being transient erythema (5.2%) and dry skin (2.1%) (Palliyage et al., 2021). Curcumin exhibits biphasic dose-response characteristics, where low doses are beneficial but higher doses may lead to toxicity or reduced efficacy (Gupta et al., 2020). Although high-efficiency nanocarriers can enhance the efficacy of drugs, they may also increase the risk of side effects due to their prolonged retention in the body. Future studies should focus on the toxicity and long-term risks of drug combination carriers, and track efficacy and safety changes simultaneously.

Enhancing the bioavailability and stability of curcumin has become a major focus in curcumin research and is fundamental to the development of curcumin-related formulations. Current research is primarily centered on curcumin derivatives and prodrugs, pharmaceutical strategies, and combination therapies to position curcumin at its target sites, thereby improving its therapeutic efficacy. Curcumin-supported delivery systems include nanoparticles, magnetic nanoparticles, solid lipid nanoparticles, liposomes, nanostructured lipid carriers, microgels, hydrogels, biopolymer nanoparticles, micelles, phospholipid complexes, emulsions, microemulsions, nanoemulsions, and metal complexes. Through these delivery systems, curcumin’s water solubility, efficacy, stability, bioavailability, and target concentration have been markedly improved (Flory et al., 2021; Wen et al., 2023). The wide application of nanocarriers is largely attributed to their ability to penetrate biological barriers and exert therapeutic effects in the human body (Jarvis et al., 2019; Liu et al., 2022; Wang et al., 2023).

Different nano-formulations have their own characteristics and advantages. Lipid-based nanoparticles (LNPs), such as nanostructured lipid carriers (NLCs) and solid lipid nanoparticles (SLNs) (Amer et al., 2021; Kotb et al., 2023), have emerged as the most promising formulations due to their high biocompatibility, controlled release properties, significant collagen-enhancing effects, and inhibition of MMPs. Nanoemulsions (Laksmiani et al., 2022), characterized by small droplet sizes (20–200 nm), exhibit superior skin penetration and versatility in encapsulating diverse actives (e.g., curcumin, resveratrol, thymol), synergistically amplifying anti-inflammatory and antioxidant activities for clinical applications. Metal nanoparticles (Rehman et al., 2023) particularly those coated with plant extracts to reduce toxicity, offer dual protection against photoaging through combined antioxidant and UV-absorbing capabilities. While ethosomes (Sallustio et al., 2023) demonstrate strong permeation efficiency, their ethanol concentration requires optimization to minimize skin irritation. Nanogels, featuring high mucoadhesion and sustained release profiles (e.g., chitosan/alginate gels achieving 5-day release), enhance localized therapeutic efficacy when integrated with functional materials like carbon nanosponges (Niu et al., 2023; Sideek et al., 2024).

Comparative analyses highlight liposomes and microemulsions as research hotspots for their exceptional permeability, whereas SLNs and nanogels excel in sustained release and localized treatment. Critical challenges remain: LNPs require resolution of long-term storage stability and scalable manufacturing processes (Kotb et al., 2023); nanoemulsions demand balanced surfactant safety to prevent skin barrier disruption and standardized clinical efficacy metrics (e.g., long-term validation of photoaging biomarkers). Nanogel formulations necessitate strict pH control (5.7–6.2) to avoid cutaneous discomfort. Future directions emphasize clinical translation of curcumin nanoformulations and exploration of personalized designs to achieve efficient, safe anti-photoaging therapies.

By exploiting the unique properties of these nanocarriers, the full therapeutic potential of curcumin spans a wide range of applications, including its antibacterial, antioxidant, anti-inflammatory, neuroprotective, and anticancer properties. These diverse therapeutic roles highlight curcumin’s versatility in various medical interventions—can be further realized, benefiting clinical outcomes (Ferguson et al., 2021; Dehzad et al., 2023). Various nanoparticle-based approaches have been studied for the effective delivery of curcumin in different skin diseases. For clarity, selected case studies of topical curcumin delivery have been compiled in Table 1 in dermatological conditions.

**TABLE 1:** Summary of case studies on topical administration of curcumin.

	Components incorporated	Formulation developed	Characterization techniques	Model	Inference	Ref
1	Cur/ZnO NPs	Nanofibers	Scanning Electron Microscope, X - Ray Diffraction and Fourier Transform Infrared Spectroscopy (SEM, XRD and FTIR)	Rats	Cur/Zinc Oxide/Polyvinyl Alcohol (ZnO/PVA) nanofibers showed the best wound healing effect in rats: by Day 12, the wound diameter was significantly decreased from 1.000 cm to 0.075 cm, achieve 92.5% wound contraction. It has stronger antioxidant activity, 2,2 -Diphenyl-1-picrylhydrazyl (DPPH) free radical scavenging rate of 81.4%. *In vitro*, it showed good antibacterial activity against *E. coli* and *Staphylococcus aureus*, with inhibition zones of 7 mm and 4 mm, respectively, and no cytotoxicity against L929 cells.	Nemati et al. (2024)
2	Curcumin, succinic anhydride, deoxycholic acid	Nanomicelles	Proton Nuclear Magnetic Resonance (H-NMR), FTIR, and XRD	Zebrafish	Curcumin, loaded in amphiphilic chitosan micelles (91.7% entrapment, 196.4 nm), showed markedly improved water solubility and stability. Compared to free curcumin, antioxidant activity was enhanced, with DPPH scavenging reaching 85.1% vs. 45.6% at 20 μg/mL. The micelles sustained over 80% activity across concentrations, confirming effective radical scavenging.	Chen et al. (2024)
3	Chitosan, reduced graphene oxide, curcumin, papain, collagen peptide	Nanocomposites	FTIR, Dynamic Light Scattering (DLS), XRD, SEM	Rats	The results showed that the anti-inflammatory and cell viability of Casein Plastic/Reduced Graphene Oxide/Cellulose Propionate/Curcumin/Polyamide (CS/RGO/CP/Cur/PA) were improved by 99.7% and 395%, respectively, which was higher than other methods. Animal experiments in rats showed that CS/RGO/CP/Cur/PA increased wound healing by 70%.	Elhami et al. (2024)
4	Curcumin, methylacrylated gelatin, dopamine, Zinc-doped hollow mesoporous cerium oxide	Nanoparticles	SEM, FTIR	Sprague Dawley male rats	The GeIMD-Cur@ZHMCe hydrogel exhibit potent antibacterial activity (killing rates >70% for *E. coli* and >80% for *S. aureus*), antioxidant properties (ROS scavenging >80%), and anti-inflammatory effects (reduced IL-1β/IL-6 expression). In a rat chronic wound model, it achieved 98.5% ± 4.9% healing within 14 days, outperforming controls by promoting neovascularization and acid-responsive Cur release (>70% in 60 h under acidic conditions).	Zhao et al. (2024)
5	Curcumin, geranium oil, Tween 80, propylene glycol	Microemulsion	Zeta, HI 2210 Hanna	Rat with carrageenan-induced paw edema (*ex vivo* rat skin and HEPG2 cells *in vitro*)	Among the several formulations with different compositions of geranium oil and Tween80/propylene glycol, the formulation with the highest amount of oil (20%) afforded the fastest skin permeation *ex vivo*: a flux of 130.9 μg/cm2/h and a lag time of 0.08 h. Enhanced antioxidant activity compared to pure curcumin. *In vivo*, the emulsion reduced inflammation more effectively than pure curcumin, with reductions of 93% versus 32% at 6 h.	Hassan et al. (2024)
6	Citral, Nerolidol, Eucalyptol, Curcumin, Soya phosphatidylcholine, Carbopol 934, Ethanol Triethanolamine, Methanol	Nanoinvasives	Nano Zeta Sizer, Transmission Electron Microscope (TEM), FTIR-ATR	Balb/c mouse model of psoriasis (and *ex vivo* porcine ear skin)	Curcumin, encapsulated in citral-based invasomes (85.8% entrapment, 302 nm), showed 3*enhanced skin permeation vs. conventional gel. In psoriatic mice, the formulation resolved symptoms within 10 days by suppressing keratinocyte hyperproliferation and inflammation via NF-κB/STAT3 inhibition, proving invasomes as a potent transdermal delivery system for curcumin’s therapeutic efficacy.	Kumar and Sahoo (2023)
7	Curcumin, Fusidic Acid, Tween 80, Lecithin, Carbopol 934	Nanogel, Mixed Micelles	Zeta, TEM, Differential Scanning Calorimetry (DSC), FTIR	Sprague-Dawley rat model of acne (and *ex vivo* rat skin)	The permeability of Mixed Curcumin-Fusidic Acid-Micelles (Cur-FA-MM) nanogels was increased by 2 times, and the skin deposition (Cur: 562.07 μg/CM2, FA: 405.47 μg/CM2) was significantly higher than that of ordinary gels. Ear thickness was reduced by 70% in the Cur-FA-MM group, and histopathology showed normalization of the epidermis with minimal inflammatory cell infiltration. Hybrid micellar nanogels extend drug residence time by sustained release and enhanced permeation.	Abdel-monem et al. (2023)
8	Copper sulfide, curcumin, methanol, F127-CHO micelles	nanoparticle	SEM, XRD	SD rats, Human umbilical vein endothelial cells	CUR/CuS@F127 hydrogel reached 53.1°C under 808 nm NIR in 5 min and showed strong antioxidant and antibacterial activity. *In vivo*, it achieved 95.3% wound closure by day 10 with enhanced collagen deposition and angiogenesis.	Jia et al. (2023)
9	Polyamide 6, hyaluronic acid, and curcumin	Nanofibers	FTIR	Male rats	The optimized PA6/HA/HNT@Cur nanofibrous membrane with 1 wt% HNT@Cur exhibited controlled pH-sensitive curcumin release, achieving 98% release at acidic pH (5.4) and 85% at physiological pH (7.4), alongside strong antibacterial activity against gram-positive/negative pathogens and 70% antioxidant activity. *In vivo* studies demonstrated superior wound healing, with 81% wound contraction after 14 days compared to 40% in untreated controls, and enhanced tissue regeneration confirmed by histological analysis.	Shakiba et al. (2023)
10	Curcumin, Labrasol, Cremophor RH 40, Transcutol P, isopropyl myristate	Self-nano	DLS, Polydispersity Index (PDI)	HaCaT, Caco-2	The SN/MEDDS achieve high drug loading efficiency (93.11%–99.12%) and sustained thermodynamic stability. SNEDDS demonstrated superior *in vitro* curcumin release (over 80% at 180 min) and antioxidant activity (52.66% DPPH scavenging) compared to SMEDDS and pure curcumin. Additionally, Labrasol-based SNEDDS reduced TNF-α and IL-1β levels below 60% in inflamed cells, showing enhanced anti-inflammatory effects and non-cytotoxicity at 5% w/v.	Józsa et al. (2022)
11	Curcumin, Graphene oxide (GO) powder, alginate (ALG) solution in double-distilled water (ddH2O)	Nanosheet	SEM, Atomic Force Microscopy (AFM)	HBEpc, HPV-negative VA, United States	The CUR/GO hybrid hydrogel showed improved thermal stability (20% vs. 30% weight loss) and water resistance compared to pure hydrogels. At 2.5% loading, it reduced GO toxicity in normal cells while effectively killing squamous cell carcinoma cells (SCC) cells, with sustained CUR release (∼50% over 96 h) via π-π interactions, indicating potential for localized SCC therapy.	Madeo et al. (2022)
12	Curcumin, carboxymethyl guargum, reduced graphene oxide	Nanocomposite	SEM, XRD, Thermogravimetric Analysis (TGA), FTIR	Rabbits	The nanocomposites achieved complete wound closure within 48 h due to the proliferation of 3T3-L1 fibroblast cells and facilitated controlled drug release. Moreover, *in vivo* studies indicated that the CMGG nanocomposite, which combines reduced graphene oxide with curcumin, has significant potential for wound healing.	Orsu et al. (2021)
13	Curcumin, chitosan, PVA, nano silver	Nanocomposite	Curcumin, l-lactic acid, citrate siloxane, polydopamine	Mice	The PPCP scaffold achieved 93% bacterial killing, 94% antioxidant activity, and 46.4°C photothermal heating, enabling synergistic tumor suppression and wound healing. It accelerated tissue repair with 74.5% curcumin release, enhanced collagen deposition, and reduced inflammation (TNF-α/IL-6) while maintaining biocompatibility.	Xi et al. (2020)
14	Curcumin, ZnO Nps	Nanoconjugates	AFM, DLS, FTIR, Zeta	Female Sprague-Dawley rats	Cur-Zno Nanocomposite had strong antioxidant activity (82.6% ± 3.3%), and the wound healing rate was 99.2%. *In vivo*, topical application resulted in 96% wound contraction on day 14, accompanied by enhanced collagen deposition and reepithelialization.	Aslam et al. (2022)
15	Curcumin	Nanosuspensions	XRD, Zeta, Malvern Panalytical Ltd., High - Performance Liquid Chromatography (HPLC), DLS	*Ex vivo* porcine ear model	Curcumin nanocrystals exhibited the highest skin penetration with a cumulative amount of 102.4 ± 12.2 μg/cm^2^ and a flux of 3.0 ± 0.7 μg/cm^2^/h. *In vivo*, nanocrystals significantly reduced UV-induced skin inflammation, achieving a 68.4% reduction in ear thickness.	Eckert et al. (2021)
16	Curcumin, chitosan	Nanoparticulate	HPLC	Human Dermal Fibroblast-Adul	Metal Matrix Composite Scaffolds-Curcumin NPs showed sustained curcumin release of 82.3% over 72 h and high cellular uptake. *In vivo*, they achieved 95.2% wound closure by day 14 and significantly promoted collagen deposition and re-epithelialization in burn-injured skin.	Basit et al. (2020)
17	Curcumin	Nanostructured Lipid	SEM, AFM, XRD	Rat	The drug release of NLC within 24 h was found to be 60.2% ± 0.45%, indicating a sustained release pattern. *In vitro* permeation study showed a good permeation flux (0.453 ± 0.76 μg/cm)2. H) and retention of CUR in the skin epidermis (60.2% ± 0.45%).	Kesharwani et al. (2020)

However, nanoparticles do have some inherent drawbacks, which vary across different environments. Although liposomal nanoparticles are promising for improving drug delivery and increasing bioavailability, they encounter several significant challenges. One major issue is their quick removal from the body, which can reduce their effectiveness. Additionally, maintaining sterility is a concern, as any contamination could lead to complications. There’s also the risk of the drug unintentionally leaking from the nanoparticles, which could affect treatment outcomes. (Barenholz, 2012; Allen and Cullis, 2013; Patil and Jadhav, 2014; Inglut et al., 2020; Gbian and Omri, 2022). The cytotoxicity of nanoparticles (NPs) hinges on multiple factors. Key mechanisms include direct physical damage to cellular structures, toxic ion release, and ROS-induced oxidative stress (Wang et al., 2017; Yu et al., 2020). Physicochemical properties critically influence toxicity: smaller NPs (<50 nm) exhibit higher reactivity due to increased surface area (Kou et al., 2018; Wolnicka-Glubisz et al., 2015), while shape determines cellular interactions—spherical NPs generally induce lower toxicity than nanotubes or rods. However, some studies have also found that spherical nanoparticles are more toxic than rod-shaped nanoparticles (Samei et al., 2019; Zhao et al., 2013). Aggregation status, surface charge, and wettability further modulate toxicity, with higher wettability accelerating degradation and protein adsorption (Li et al., 2019). Surface functionalization via biocompatible coatings mitigates adverse effects (Nguyen et al., 2013). Dose-dependent cytotoxicity is pronounced, as high concentrations disproportionately reduce cell viability, and even low doses of small NPs (e.g., 18 nm) trigger significant toxicity (Gandamalla et al., 2019; Kim I-Y. et al., 2015).

Currently, extensive research has been conducted on curcumin nanocarriers for the treatment of skin-related diseases. Among these studies, the application of curcumin nanocarriers to promote wound healing is the most prevalent, followed by research into their use for inflammatory diseases and photoaging. Local delivery via nanocarriers aims to enhance curcumin absorption and efficacy within the skin. While existing studies confirm the effectiveness of curcumin nanocarriers in treating skin conditions, several challenges remain. Despite nanotechnology’s potential to improve curcumin’s skin penetration, achieving adequate deep-layer penetration, particularly for treating more severe skin lesions, remains a challenge. Most current research is limited to *in vitro* and animal studies, and further clinical trials are needed to bridge the gap between animal models and human application. Additionally, curcumin’s susceptibility to degradation under light, heat, and varying pH conditions necessitates stable nanocarriers to protect its active components. Further investigation into the long-term stability and sustained efficacy of these nanocarriers is essential.

Collectively, the focus should be on enhancing the performance of nanocarrier systems to ensure their safety and effectiveness in clinical applications. Addressing current challenges, developing more cost-effective and scalable nanocarrier formulations, and advancing the broader application of curcumin nanocarriers are crucial steps forward.

## 5 Discussion

The document and associated literature collectively emphasize curcumin’s multifaceted role in combating skin photoaging. The following points highlight its mechanisms and applications.

First, the mechanism, curcumin exerts anti-photoaging effects primarily through its antioxidant and anti-inflammatory properties. It reduces ROS generation, inhibits MMPs, and modulates key signaling pathways such as NF-κB, MAPK, and Nrf2. It also promotes collagen synthesis and reduces UV-induced apoptotic damage in fibroblasts. Meanwhile, Topical and systemic applications of curcumin have shown promising results in mitigating UV-induced damage (Adusumilli et al., 2021). Novel delivery systems, such as nanoparticles and nanomicelles, significantly enhance its photoprotective effects by improving bioavailability and stability. Studies on curcumin derivatives, such as chlorin e6-curcumin conjugates and chemically modified curcumins (Goenka et al., 2021), reveal enhanced efficacy in reducing MMP expression and improving collagen synthesis.

The anti-photoaging effects of curcumin stem from its multidimensional molecular regulatory network. In terms of antioxidant defense, it not only directly scavenges free radicals (Singh et al., 2011) but also activates the Nrf2/ARE axis (Ashrafizadeh et al., 2020), driving the expression of key enzymes such as glutathione S-transferase and catalase to systemically alleviate ROS accumulation. For inflammatory cascades, curcumin inhibits NF-κB nuclear translocation (Zinatizadeh et al., 2021) and blocks MAPK phosphorylation (Lee et al., 2020), significantly reducing the release of inflammatory mediators like TNF-α and IL-6. Additionally, the expression of core photoaging drivers MMP-1/3 is suppressed through dual mechanisms: antagonizing AP-1 transcriptional activity and interfering with NF-κB signaling (Lee et al., 2012; Vicentini et al., 2011), thereby mitigating collagen degradation. Notably, curcumin activates 5′-adenosine monophosphate-activated protein kinase (AMPK) and inhibits mTOR (Zuo et al., 2021), remodels autophagy homeostasis to eliminate UV-induced damaged proteins, and regulates the Bcl-2/caspase-3 balance (Liu et al., 2018) and CD39/CD73-adenosine pathway (Manica et al., 2024), achieving a precise balance between anti-apoptosis and pro-repair. At the same time for the regulation of microorganisms also play a key role. This multi-target synergy ultimately preserves skin structural integrity and delays photoaging phenotypes.

The clinical application of curcumin faces multiple challenges, including its inherent low water solubility, low bioavailability due to chemical instability, insufficient penetration capacity in the deep skin, and high toxicity, and the existing studies are mostly limited to *in vitro* and animal experiments, the lack of large-scale clinical validation. Current nanoparticle formulations (such as lipid nanoparticles, nanoemulsion, metal complexes, etc.) have significantly improved the stability, targeted delivery efficiency, and local concentration of curcumin through encapsulation technology, among them, lipid carriers (such as SLNSNLCS) have become a research hotspot due to their high Biocompatibility and controlled release properties, nanoemulsion enhances skin penetration due to the advantage of small particle size, and functionalized metal nanoparticles can synergize antioxidant and photoprotection. However, the large-scale production, long-term storage stability, potential cytotoxicity (such as size-dependent oxidative stress), and clinical safety of nanoformulations still need to be further optimized.

Therefore, the future needs to be further explored to fill the current research blind spot. Stimuli-Responsive Formulations: Development of pH- or temperature-responsive drug delivery systems for targeted application (Hwang et al., 2013). Develop combination drug therapies: Combine curcumin with other antioxidants (like resveratrol) or photoprotective agents to boost its effectiveness. Regarding clinical trials, conduct large-scale randomized trials. We do this to establish standard dosages and verify long-term safety. Cost-effective production: Scale up advanced formulations. Take into account both price and accessibility to increase the likelihood of further application in treatment.

The research underscores curcumin’s remarkable potential as an anti-photoaging agent, with its ability to modulate key molecular pathways, reduce inflammation, and protect against UV-induced skin damage. However, its clinical application is hindered by bioavailability and stability challenges, which are being addressed through innovative formulations and emerging technologies. Future efforts should focus on enhancing its clinical validation, optimizing delivery systems, and ensuring cost-effective scalability. By overcoming these barriers, curcumin can be positioned as a cornerstone in the management of skin photoaging and related dermatological conditions.

## 6 Conclusion and future prospects

In summary, curcumin demonstrates remarkable efficacy in combating photoaging. Its multifaceted mechanisms primarily involve the regulation of relevant signaling pathways to exert antioxidant and anti-inflammatory effects, alongside modulation of MMP activity and influence on autophagy and apoptosis processes. These integrated actions contribute to its effectiveness in reducing wrinkle formation and mitigating skin hyperpigmentation.

Current research has positioned curcumin-based formulations as a focal point in this field. This article provides a concise overview of the intrinsic mechanisms and signaling pathways underlying photoaging, while summarizing curcumin’s key molecular targets in anti-photoaging interventions. Furthermore, it reviews recent advancements in curcumin nanoparticle delivery systems within dermatology, particularly their applications in photoaging, inflammatory disorders, and wound healing. Although existing studies on curcumin nanoparticle delivery systems are substantial, higher-quality research remains imperative to address challenges in dermatological applications.

Future research should prioritize several critical directions. First, optimizing nanoparticle delivery systems to enhance targeted delivery capabilities and rigorously evaluating the safety and efficacy profiles of curcumin formulations are essential for clinical translation. At present, although there are a large number of curcumin preparations related research, but its long-term safety needs to be further studied. Optimizing nanoparticle delivery systems enhances curcumin’s bioavailability and tissue targeting while minimizing side effects. Strategies like ligand conjugation, stimuli-responsive release, and stealth coatings improve efficacy. Clinical translation requires rigorous safety testing, scalable production, and standardized regulatory evaluation to ensure therapeutic viability.

Second, investigating the synergistic mechanisms between curcumin and other antioxidants (e.g., vitamin C, resveratrol) at the molecular level could maximize anti-photoaging outcomes through optimal combinatorial strategies. The combination can further enhance the efficacy of the drug, which has also been explored in recent years. In an Alzheimer’s disease (AD) model, curcumin combined with a coenzyme Q10 analog (mitoxantrone) was more effective than single agents in inhibiting AΒ aggregation and tau phosphorylation. (Xie et al., 2024). The combination of curcumin and vitamin C reflected an additive effect demonstrated by a significant decrease in malondialdehyde (p < 0.05) (Khudair and Al-Gareeb, 2024). These findings highlight the anti-inflammatory, antioxidant potential of combined antioxidants.

Third, emerging evidence highlights curcumin’s potential in skin barrier repair and hydration maintenance, warranting mechanistic exploration using advanced skin models. Finally, developing cost-effective nanoparticle production methods will be crucial to facilitate broader clinical implementation of curcumin-based nanotherapeutics. Curcumin promotes skin repair by modulating inflammatory pathways and enhancing hydration through hyaluronic acid synthesis. Nanoemulsion gels containing curcumin, resveratrol, and thymoquinone improve skin hydration and barrier function in psoriasis (Khatoon et al., 2021). Curcumin plus piperine significantly improves body composition by increasing muscle mass in patients with mild to moderate IBD (da Paz Martins et al., 2025). Commercial applications like Relispray^®^ (a turmeric-based spray bandage) leverage curcumin’s wound-healing properties (Lagoa et al., 2025). These effects position curcumin as a promising ingredient in dermatological formulations for inflammatory skin conditions and cosmetic hydration therapies.

It is worth noting that skin-specific factors such as pH, temperature, and photosensitivity significantly influence drug efficacy. Advanced delivery systems enhance curcumin’s topical efficacy through stimuli-responsive mechanisms. It is of great significance to develop stimuli-responsive drug delivery systems for targeted skin applications. pH/Enzyme-Responsive Systems: Chitosan-curcumin nanogels release drugs in acidic wound environments (Sideek et al., 2024). Light-Activated Nanocarriers: Gold nanoparticle-curcumin conjugates enable photothermal therapy for psoriasis (Yu et al., 2021). Microneedle Patches: Dissolvable microneedles with SLNs improve transdermal penetration (Prabhu et al., 2022). These innovations enable precise, localized delivery, minimizing systemic side effects while maximizing therapeutic benefits for dermatological and cosmetic applications.

In conclusion, curcumin emerges as a potent anti-photoaging agent through its multifaceted mechanisms targeting oxidative stress, inflammation, and cellular repair processes. While current nanoparticle delivery systems show promising advancements in dermatological applications, further optimization of targeted delivery, rigorous safety evaluations, and exploration of synergistic combinations with other antioxidants remain critical for clinical translation. Future research should prioritize developing cost-effective production methods and advanced skin-specific formulations to fully harness curcumin’s therapeutic potential in skin health and rejuvenation.
